# Change in the distribution of *Streptococcus pneumoniae* serotypes causing invasive pneumococcal disease among pediatric and adult patients in Chile between 2016 and 2023

**DOI:** 10.3389/fmicb.2026.1819434

**Published:** 2026-05-14

**Authors:** Angélica R. Bravo, Margarita Fuentes-Díaz, Antonio Cárdenas, Jonatan J. Carvajal, Carolina Jaldin, Daniza Jaldin, Marcia A. Pereyra, Valeria García, Juan Araya-Orellana, Maykol Araya-Castillo, Freddy Roach-Poblete, Francisca Valdivieso Rios, Paz B. Tabilo-Valenzuela, Evelyn L. Jara, Susan M. Bueno, Alexis M. Kalergis, Margarita K. Lay

**Affiliations:** 1 Department of Biotechnology, Faculty of Marine Sciences and Biological Resources, Universidad de Antofagasta, Antofagasta, Chile; 2 Cellular Microbiology and Photodynamics Laboratory, Center for Applied Medical Sciences, Faculty of Medicine and Health Sciences, Central University of Chile, Santiago, Chile; 3 Department of Medical Sciences, Faculty of Medicine and Dentistry, Universidad de Antofagasta, Antofagasta, Chile; 4 Pediatric Service, Dr. Leonardo Guzmán, Regional Antofagasta Hospital, Antofagasta, Chile; 5 Center for Research in Immunology and Biomedical Biotechnology of Antofagasta, Universidad de Antofagasta, Antofagasta, Chile; 6 Microbiology Area, Clinical Laboratory, Dr. Leonardo Guzmán Regional Antofagasta Hospital, Antofagasta, Chile; 7 Microbiology Laboratory, Dr. Luis Calvo Mackenna Hospital, Santiago, Chile; 8 Department of Pharmacology, Faculty of Biological Sciences, University of Concepción, Concepción, Chile; 9 Millennium Institute on Immunology and Immunotherapy, Facultad de Ciencias Biológicas, Pontificia Universidad Católica de Chile, Santiago, Chile; 10 Departamento de Endocrinología, Facultad de Medicina, Pontificia Universidad Católica de Chile, Santiago, Chile; 11 Millennium Institute of Immunology and Immunotherapy, Universidad de Antofagasta, Antofagasta, Chile

**Keywords:** epidemiology, invasive pneumococcal disease, serotype replacement, *Streptococcus pneumoniae*, vaccination

## Abstract

**Introduction:**

*Streptococcus pneumoniae* remains a leading cause of invasive pneumococcal disease (IPD), particularly among young children and older adults. Although pneumococcal conjugate and polysaccharide vaccines have reduced IPD caused by vaccine-included serotypes, the emergence of non-vaccine serotypes has increasingly offset these benefits. Updated regional data are essential to inform vaccine strategies in Latin America.

**Methods:**

We conducted a retrospective, hospital-observational, cross-sectional study of IPD cases diagnosed between 2016 and 2023 at two public hospitals in Chile. Clinical records and laboratory reports were reviewed to collect demographic, clinical, vaccination, and serotype data from patients with confirmed IPD. Descriptive statistics were used to summarize qualitative variables as frequencies and quantitative variables using measures of central tendency and dispersion.

**Results:**

Non-vaccine *Streptococcus pneumoniae* serotypes predominated in cases of IPD in children and adults during the study period. In the pediatric population, the most frequently isolated serotypes were 19A, 24F, 6C, and 3, while in adults, serotypes 3, 23A, 23B, 19A, and 6C were identified. Of these, only serotypes 3 and 19A are included in currently available vaccines. Furthermore, vaccination coverage was high in children (75.5%) but very low in adults (6.3%). Mortality was observed exclusively in adults, reaching 20.4%.

**Discussions:**

IPD in Chile during 2016–2023 was driven mainly by non-vaccine serotypes and was associated with substantial mortality among adults. These findings suggest a replacement of vaccine serotypes by non-vaccine serotypes and reveal critical deficiencies in the protection of adults against serotypes that cause IPD. Updated serotype surveillance and reassessment of pneumococcal vaccine strategies, including adult immunization policies, are warranted to improve prevention of severe pneumococcal disease in Chile and similar settings.

## 1 Introduction

*Streptococcus pneumoniae* (pneumococcus) is a Gram-positive, alpha-hemolytic diplococcus that causes a wide range of diseases (Gierke et al., 2021). *S. pneumoniae* can lead to invasive pneumococcal disease (IPD), particularly dangerous for children, older adults, and immunocompromised individuals. In the infant population, about one million deaths from IPD are recorded worldwide each year (Bragason et al., 2025). This, along with the increase in antimicrobial resistance, makes pneumococcus a serious and rapidly growing global health challenge (Nazir et al., 2025; Steve et al., 2026; WHO Expert Committee on Biological Standardization, 2013).

There are over 100 pneumococcal serotypes that differ in the polysaccharide composition of their capsules (Eichner et al., 2025). Although prevention and vaccination strategies are available, they are not fully effective against all *S. pneumoniae* serotypes because natural variation can lead to the emergence of new serotypes (Feikin et al., 2013; Harboe et al., 2014; Johnson et al., 2024; Paton and Trappetti, 2019; Weinberger et al., 2019; Zhou et al., 2017).

Antibodies against *S. pneumoniae* capsular polysaccharide antigens provide serotype-specific protection against pneumococcal disease (Nurse-Lucas et al., 2016; Thong et al., 2023). Two types of anti-pneumococcal vaccines are currently available in Chile: one based on purified capsular polysaccharides (PPSV) and another based on polysaccharides conjugated to a carrier protein (PCV). The PPSV vaccine protects against 23 serotypes [Pneumovax 23, Merck Sharp & Dohme (MSD) Corp.] responsible for about 60–76% of IPD (1, 2, 3, 4, 5, 6B, 7F, 8, 9N, 9V, 10A. 11A, 12F, 14, 15B, 17F, 18C, 19A, 19F, 20, 22F, 23F, and 33F) (Prunas et al., 2024; Sohail et al., 2018). It is recommended for older adults, immunocompromised individuals, and children aged two or older. Conversely, the PCV vaccine protects against 13 serotypes (Prevenar 13, Pfizer), covering nearly 65% of IPD cases among children younger than 5 years old (serotypes 1, 3, 4, 5, 6A, 6B, 7F, 9V, 14, 18C, 19A, 19F, and 23F) (Bruhn et al., 2017; Pilishvili et al., 2010; Sørensen, 1993). In addition, PCV13 is administered at 2, 4, and 12 months (three doses), while PPSV23 is administered only in a single dose in adults over 65 years of age and immunocompromised persons (Calvo et al., 2020). Using PCVs in infants has significantly reduced IPD prevalence and provided indirect benefits for unvaccinated groups (Moberley et al., 2013; Savulescu et al., 2017; von Gottberg et al., 2014). Although the impact on reducing hospitalizations and pneumonia-related mortality remains controversial, several studies support its cost-effectiveness in the United States and Europe (Blamey, 2014; Weinberger et al., 2011). IPD caused by vaccine serotypes is partially offset by increases caused by non-vaccine serotypes, a phenomenon known as serotype replacement (Muhammad et al., 2013; Paton and Trappetti, 2019; von Gottberg et al., 2014). The overall benefit of vaccines depends on both the extent of IPD reduction caused by vaccine serotypes and the increase in IPD caused by non-vaccine serotypes (Nieto Guevara and Guzman-Holst, 2021; Paton and Trappetti, 2019). Currently, there are other vaccines available worldwide: the PCV15 (Merck & Co.), which offers protection against the PCV13 vaccine serotypes plus the serotypes 22F and 33F, used especially in the European Union (EU), Europe and Asia-Pacific; and the PCV20 (Wyeth Pharmaceuticals LLC), which covers the PCV13 serotypes plus serotypes 8, 10A, 11A, 12F, 15B, 22F and 33F, used mainly in Europe, the EU, Asia-Pacific and Latin America (Mexico and Argentina) (Gierke et al., 2021; Weinberger et al., 2010).

In Chile, the PCV10 vaccine (Synflorix, GSK), which protects against 10 pneumococcal serotypes (1, 4, 5, 6B, 7F, 9V, 14, 18C, 19F, and 23F), was added to the National Immunization Program (PNI, for its acronym in Spanish) of Chile in 2011. In November 2017, PCV13 replaced PCV10 and has been part of the PNI since then (Weinberger et al., 2011). Chile participates in SIREVA (for its Spanish acronym), a Latin American initiative that supports laboratory-based surveillance of IPD (Ganaie et al., 2020). Additionally, the Public Health Institute (ISP, for its acronym in Spanish) prepares periodic regional and national reports on IPD surveillance. However, these reports often lack a detailed analysis of relevant information on IPD patients. Therefore, studying the serotype distribution of *S. pneumoniae* among patients diagnosed with IPD is essential for evaluating the effectiveness and efficacy of PCV and PPSV vaccines in these patients (Bruhn et al., 2017; Sharew et al., 2024; WHO Expert Committee on Biological Standardization, 2013).

This study aims to analyze the distribution of *S. pneumoniae* serotypes causing IPD in pediatric and adult patients, circulating from January 2016 to December 2023, in two public hospitals in the cities of Antofagasta and Santiago, Chile. Serotype distribution was correlated with vaccination status, socioeconomic data, diagnosis, underlying diseases (risk factors), and antibiotic susceptibility to provide more robust and detailed information about the IPD population in Chile.

## 2 Materials and methods

This is a retrospective, hospital-observational, cross-sectional study based on data collected from two public hospitals in Chile between 2016 and 2023.

Clinical and microbiological data were collected from medical records and laboratory surveillance databases. The reporting of this study follows the Strengthening the Reporting of Observational Studies in Epidemiology (STROBE) guideline (Vandenbroucke et al., 2007).

### 2.1 Settings and participants

This study was conducted in two public hospitals located in different regions of Chile: the Dr. Luis Calvo Mackena Pediatric Hospital in Santiago, and the Dr. Leonardo Guzman Regional Hospital in Antofagasta. These institutions serve as referral and surveillance centers for the pediatric and adult populations within their respective health networks. In Chile, IPD transmission occurs primarily during the winter (May to August).

All patients with a confirmed laboratory diagnosis of IPD, defined as the isolation of *S. pneumoniae* from sterile sites (blood, cerebrospinal fluid, pleural, peritoneal, or synovial fluid), between January 2016 and December 2023, were included regardless of vaccination status. Cases without microbiological confirmation, with incomplete clinical records, or missing information on key variables (age, or *S. pneumoniae* serotype) were excluded.

### 2.2 Data collection and eligibility criteria

Clinical and epidemiological data were obtained retrospectively from the medical records of patients with IPD treated at two public hospitals in Chile: the Dr. Luis Calvo Mackenna Pediatric Hospital in Santiago and the Dr. Leonardo Guzmán Regional Hospital in Antofagasta, between 2016 and 2023.

Collected data included demographic characteristics (age, gender, country of birth), clinical diagnoses (underlying comorbidities such as diabetes, chronic respiratory disease, immunosuppression, chronic liver disease), Intensive Care Unit (ICU) admission, length of hospital stay, in-hospital mortality, and antibiotic susceptibility. Antimicrobial Susceptibility Testing (AST): AST was conducted using two methods: E-test gradient diffusion (bioMérieux) for the 2016–2020 period (penicillin, cefotaxime, and vancomycin) and the BD Phoenix™ M50 automated system (SMIC/ID-8 panel) from 2020 onwards for an expanded antimicrobial panel. In both periods, MIC values were interpreted according to contemporaneous CLSI M100 breakpoints, applying site-specific criteria (meningeal vs. non-meningeal) for β-lactams. All isolates were referred to the Public Health Institute (which stands for ISP in Spanish) of Chile for serotyping and confirmatory AST, ensuring external quality control and longitudinal comparability (Lewis, 2024).

Vaccination status was obtained from the records of the Chilean National Immunization Program when available (Weinberger et al., 2011). The immunization status of each patient was recorded as “vaccination status: complete, incomplete, or not vaccinated”. Specifically, the complete vaccination schedule for PCV10 and PCV13 consists of three doses, administered at 2, 4, and 12 months of age. In contrast, the PPSV23 vaccine consists of a single dose for adults over 65 years of age and immunocompromised individuals.

Cases whose clinical records or laboratory data could not be accessed were excluded from the analyses. Age was analyzed as a continuous variable and categorized into five groups (> 1, 1–4, 5–17, 18–64, and ≥ 65 years). Hospitalization duration was summarized using means and standard deviations or medians and interquartile ranges, depending on the data distribution.

Prior to analysis, the dataset was screened for completeness. Missing data were identified in key clinical variables, including ICU admission, mortality, and nationality. To handle these missing values, we performed sensitivity analyses (point 2.5 in this section).

To reduce selection bias, all IPD cases confirmed during the study period were included. Information bias was minimized by having two independent researchers review medical records; disagreements were resolved through consensus with M.K.L.

### 2.3 Detection and serotyping of *S. pneumoniae*

This study delineated invasive pneumococcal disease (IPD) as the isolation of *Streptococcus pneumoniae* from normally sterile sites such as blood, cerebrospinal fluid, or pleural fluid. Serotype data were collected through the National Laboratory Surveillance System of the ISP in Chile. Diagnosis was based on bacterial isolates obtained via conventional culture techniques from clinical specimens. Capsular serotyping was performed using the modified Quellung reaction (Neufeld reaction), which represents the standard method for pneumococcal identification in Chile. This procedure involves examining the pure culture isolate with specific polyclonal antisera provided by the Statens Serum Institute in Copenhagen, Denmark. A positive result is indicated by optical microscopy, demonstrating swelling of the bacterial capsule, allowing for precise serotype determination (Habib et al., 2014).

### 2.4 Ethical approval

This retrospective cross-sectional study was conducted by the University of Antofagasta (UA) in collaboration with two public hospitals in Chile: “Dr. Luis Calvo Mackenna Pediatric Hospital” in Santiago (Ethical Approval Resolution No. 1577) and “Dr. Leonardo Guzmán Regional Hospital” in Antofagasta (Ethical Approval Resolution No. 409/2022), after the Ethics Committees of each institution approved the protocol. The methods and research received informed consent from the legal guardians of children under 17 years of age. Informed consent was obtained from adult patients (over 18 years old).

Access to the data was restricted to medical personnel from each institution. The data was later anonymized using codes.

### 2.5 Statistical analysis

The data collected from the clinical records were organized and managed using Microsoft Excel. Statistical analyses were performed using SPSS (version 25.0).

A descriptive analysis was conducted to characterize the study population. Frequencies and percentages were calculated for categorical variables (sex, nationality, vaccination status, ICU admission, vital status, clinical presentation, and antibiotic susceptibility). To assess the associations between these categorical variables, Pearson’s chi-squared test or Fisher’s exact test was used, as appropriate.

For continuous variables, such as length of hospital stay, the Shapiro-Wilk test was used to evaluate data normality. Given the non-normal distribution of the data, these variables were summarized using medians and interquartile ranges (IQR), and comparisons between groups (children vs. adults) were performed using the Mann-Whitney U test.

The distribution of *S. pneumoniae* serotypes and the annual prevalence of IPD were analyzed and visualized using trend lines and bar charts to identify changing patterns during the study period (2016–2023). Statistical significance for all tests was defined as a *p* < 0.05

Sensitivity analyses were performed to assess the influence of missing data on key variables, including ICU admission, mortality, and nationality. For ICU admission, the results of the complete-case analysis were compared with outlier-imputation scenarios. Regarding mortality, since no events were observed in the pediatric group, comparisons were made using Fisher’s exact test. Outlier-imputation scenarios were also evaluated, considering missing data as either events or non-events, to estimate potential variability in the effect measures. For nationality, the sensitivity analysis was performed by reclassifying records with missing data as national (Chilean) or foreign, and comparing these scenarios with the complete-case analysis.

## 3 Results

### 3.1 Participants and clinical outcomes

The final sample included 97 patients with invasive pneumococcal disease (IPD), 49 children, and 48 adults, who were treated at two hospitals in Chile, located in different geographical regions, between 2016 and 2023. In the pediatric population, the highest proportion of IPD cases occurred in the 1–4-year-old group at 51% (25 patients), followed by the 5–17-year-old group at 32.7% (16 patients), and finally, the group of infants under 1 year of age at 16.3% (8 patients). In the adult population, the 18–64-year-old group had the most IPD cases at 75% (36 patients), while the group over 65 years of age had 25% of the cases (12 patients). The characteristics of these patients are detailed in Table 1.

**TABLE 1:** Characteristics of the 97 IPD patients of two hospitals of Chile between 2016 and 2023.

Characteristic	Category	Children *n* = 49 (%)	Adults *n* = 48 (%)	*p*-value	OR (95% CI)
Age ranges	<1	8 (16.3)			
	1–4	25 (51.0)			
	5–17	16 (32.7)			
	18–64		36 (75.0)		
	> 65		12(25.0)		
Gender	Male	27 (55.1)	31 (64.6)	0.41	0.67 (0.29–1.55)
	Female	22 (44.9)	17 (35.4)		
ICU admission	Yes	16 (34.8)	15 (55.6)	0.08*	2.34 (0.85–6.40)
	No	30 (65.2)	12 (44.4)		1.00
Discharge vital status	Alive	48 (100)	17 (63.0)	< 0.001*	Not estimable*
	Dead	0 (0.0)	10 (37.0)		–
Vaccination status	Vaccinated^a^	35 (74.5)	3 (6.7)		1.00
	Not vaccinated/incomplete schedule	12 (25.5)	42 (93.3)	< 0.001*	38.54 (9.77–229.48)
Nationality	Chilean	43 (95.6)	22 (84.6)	0.1825	3.83 (0.50–45.45)
	Foreign	2 (4.4)	4 (15.4)		1.00
Medium hospital-stay in days (interquartile distance)		7 (14)	14 (30)		
Clinical form of IPD event n, (%)					
Respiratory infections		22 (44.9)	16 (33.3)	0.018	
CNS infections		4 (8.2)	11 (22.9)		
Systemic and hematologic infections		7 (14.3)	4 (8.3)		
Febrile syndromes		8 (16.3)	0 (0.0)		
Various localized infections		4 (8.2)	3 (6.3)		
Other		1 (2.0)	1 (2.1)		
W.I.		3 (6.1)	13 (27.1)		
Underlying risk factors					
Chronic kidney disease		0 (0.0)	1 (2.1)		
Diabetes mellitus		0 (0.0)	5 (10.4)		
Drug abuse and alcoholism		0 (0.0)	3 (6.3)		
Chronic lung disease (asthma, COPD)		3 (6.1)	3 (6.3)		
Chronic liver disease		0 (0.0)	1 (2.1)		
Immunosuppression		15 (30.6)	8 (16.7)		
Malformation		4 (8.2)	0 (0.0)		
W.I.		27 (55.1)	27 (56.1)		

OR, Odds Ratio; 95% CI, 95% confidence interval. A value of 1.00 indicates the reference category for each independent variable in the regression model. *OR not estimable due to zero cell; Fisher’s exact test used.

*^a^*Children received PVC10 or PCV13 vaccines. Adults were immunized with PSSV23.

The distribution of gender was similar between children and adults, with no statistically significant differences (Fisher’s exact test, *p* = 0.41; OR = 0.67; 95% CI: 0.29–1.55).

A significant difference in mortality was observed between children and adults. No children died, while in adults, the mortality rate was 37.0% (10 cases). This difference was statistically significant (Fisher’s exact test, *p* < 0.001). The odds ratio could not be precisely estimated due to the absence of events in the pediatric group, suggesting a trend toward a higher risk of mortality in adults. The absence of infant mortality events limits the precision of the effect estimate. However, consistent results in sensitivity analyses support a notably higher mortality risk in adults. A sensitivity analysis was conducted to assess the impact of missing data on mortality estimates. In all evaluated scenarios, no deaths were observed in the reference group (children), resulting in odds ratios that are not estimable and tend toward infinity. Despite this, the association between age group and mortality remained statistically significant in all scenarios (*p* < 0.001). The confidence intervals were wide, reflecting uncertainty due to the low frequency of events and the presence of zero-value cells.

The predominant nationality was Chilean in children (95.6%) and adults (84.6%). The proportion of foreigners was higher among adults than children (15.4% vs. 4.4%). However, this difference was not statistically significant (Fisher’s exact test, *p* = 0.18). Although adults were more likely to be foreign (OR = 3.83; 95% CI: 0.50–45.43), the wide confidence interval indicates considerable uncertainty in the estimate (Table 1). Sensitivity analyses for nationality showed considerable variability depending on the assumptions applied to missing data. The observed change from a non-significant association in the primary analysis to a strong association in extreme scenarios highlights the potential impact of information bias.

A marked disparity in vaccination status was observed between the two patient groups with IPD. Among children, 74.5% (35 cases) were vaccinated, compared to only 6.7% (3 cases) of adults. This difference was highly significant (Fisher’s exact test, *p* < 0.001). Adults had a significantly higher likelihood of being unvaccinated compared to children (OR = 38.54; 95% CI: 9.77–229.48). Hospital stays also differed significantly (*U* = 665, *p* < 0.0001). In fact, children had a mean stay of 7 days (IQR: 14), while adults had a mean of 14 days (IQR: 30) (Table 1).

The distribution of clinical manifestations differed significantly between children and adults (*p* = 0.018). Children more frequently presented febrile and respiratory forms, while adults showed a higher proportion of central nervous system involvement, suggesting differences in disease severity between the groups (Table 1).

When comparing comorbidities (Table 1), immunosuppression was the most frequent risk factor in children (30.6%, 15 cases) compared to adults (16.7%, 8 cases). Furthermore, conditions such as diabetes mellitus (10.2%) and drug addiction/alcoholism (6.3%) were recorded only in adults. Chronic lung disease was present in 6.1% and 6.3% of children and adults, respectively, with 3 cases reported in each group. Although these prevalences are low, they represent a consistent clinical characteristic in both populations of patients with IPD.

ICU admission was more common in adults than in children (55.6% vs. 34.8%). However, this difference was not statistically significant (Fisher’s exact test, *p* = 0.093). Children had a lower likelihood of ICU admission compared to adults (OR = 0.43; 95% CI: 0.14–1.26), although the confidence interval included the null value. For ICU admission, the primary analysis showed a trend toward a lower probability of admission in the group of interest (OR = 0.39; 95% CI: 0.15–1.00; *p* = 0.051). In sensitivity analyses, the magnitude and significance of the association varied across scenarios. In the worst-case scenario, where all missing data ended at ICU, the association became stronger and statistically significant (OR = 0.18; 95% CI: 0.07–0.41; *p* < 0.001), while in the best-case scenario (supposing missing data did not ended at ICU), the association was completely attenuated (OR = 1.00; 95% CI: 0.44–2.30; *p* = 0.997). These findings suggest that the observed association is sensitive to how missing data are handled (Table 1).

### 3.2 Variation in IPD prevalence caused by *S. pneumoniae* in two hospitals in Chile, from 2016 to 2023

**FIGURE 1:**
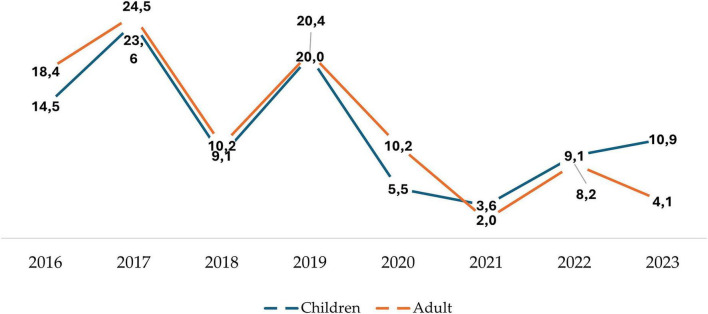
Annual percentage variation of IPD cases in adult and pediatric patients from two hospitals in Chile between 2016 and 2023. Incidence of IPD cases in percentages (y-axis), in children (blue line) and adults (red line), shown by year (x-axis). The total number of cases corresponds to the accumulated cases across all periods studied.

A notable variation in IPD prevalence caused by *S. pneumoniae* was observed in the studied population from the two studied institutions (Figure 1). In the pediatric group (blue line), the highest number of IPD cases occurred in 2017 (23.6%), followed by a second peak in 2019 (20%), a low in 2021 (3.6%), and a slight increase in 2023 (10.9%). In adults (Figure 1, red line), the pattern was similar. The highest IPD cases were recorded in 2017, at 24.5%, followed by 2019 at 20%. Afterward, there was a sharp decline, reaching a low of 2.0% in 2021. A temporary increase was observed in both groups (adults and children) in 2022.

### 3.3 Distribution of *S. pneumoniae* serotypes isolated from hospitalized patients with IPD from 2016 to 2023 in Chile

**FIGURE 2:**
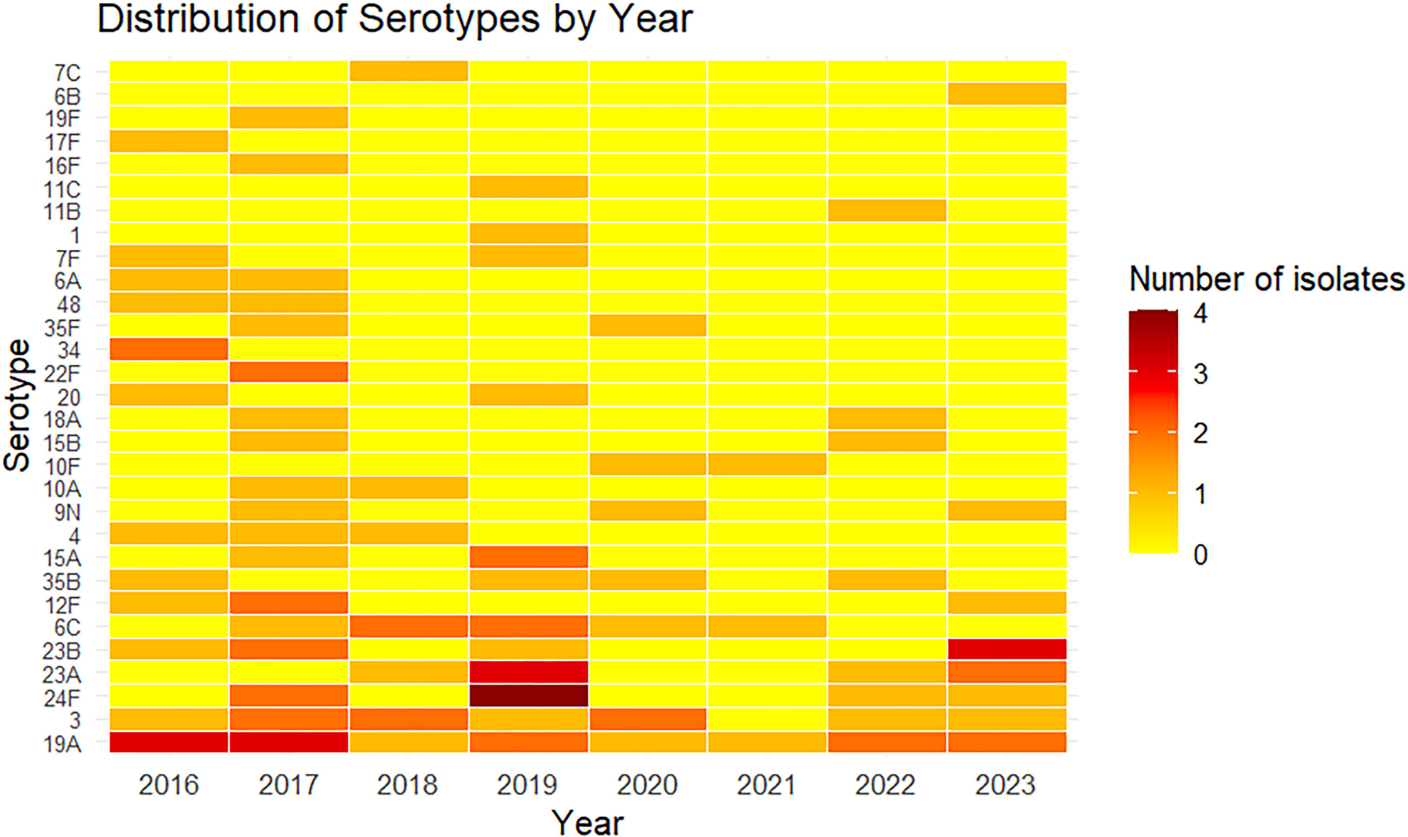
Annual distribution of pneumococcal serotypes. Color intensity reflects the number of isolates identified for each serotype in each year, ranging from yellow (low frequency) to dark red (high frequency).

A heatmap analysis was performed of the annual distribution of *S. pneumoniae* serotypes isolated from patients from two hospitals with IPD between 2016 and 2023 in Chile (Figure 2). This analysis showed significant variation in the timing of the appearance of different pneumococcal serotypes isolated from hospitalized patients with IPD during this period. Some *S. pneumoniae* serotypes remained consistently present over time, indicating a stable predominance throughout the study period. Other serotypes, however, appeared intermittently or at low levels, suggesting a constantly changing serotype distribution. The variation in color intensity over time also indicates changes in serotype prevalence, possibly reflecting alterations in epidemiological trends and selective pressures. These results highlight the coexistence of dominant *S. pneumoniae* serotypes with emerging or fluctuating ones.

**FIGURE 3:**
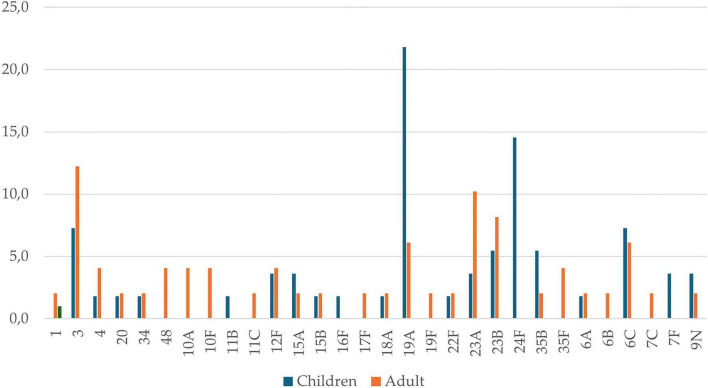
Frequency of *S. pneumoniae* serotypes isolated from IPD cases in children and adults, 2016-2023. Several serotypes (x-axis) are displayed, with age-specific patterns shown by adults (red bars) and children (blue bars). The y-axis indicates the number of cases. Statistical analysis shows significant differences in S. pneumoniae serotype distribution between children and adults with IPD (χ^2^ = 65.19, *p* > 0.001).

The analysis of *S. pneumoniae* serotype distribution was conducted separately in children and adults, revealing notable differences in serotype prevalence between the two groups (Figure 3). Serotype 19A was the most common in children, with a prevalence of 21.8%, followed by 24F at 14.5%. Other serotypes, such as 3 (7.3%), 6C (7.3%), 23B (5.5%), and 35B (5.5%), also had high frequencies across the study. In contrast, serotype 3 (12.2%) was the most frequently identified cause of IPD in adults, followed by 23A (10.2%), 23B (8.2%), and 6C and 19A, each at 6.1% (Supplementary Information S1–S3).

### 3.4 Variation in the distribution of vaccine and non-vaccine *S. pneumoniae* serotypes from 2016 to 2023 in Chile

**FIGURE 4:**
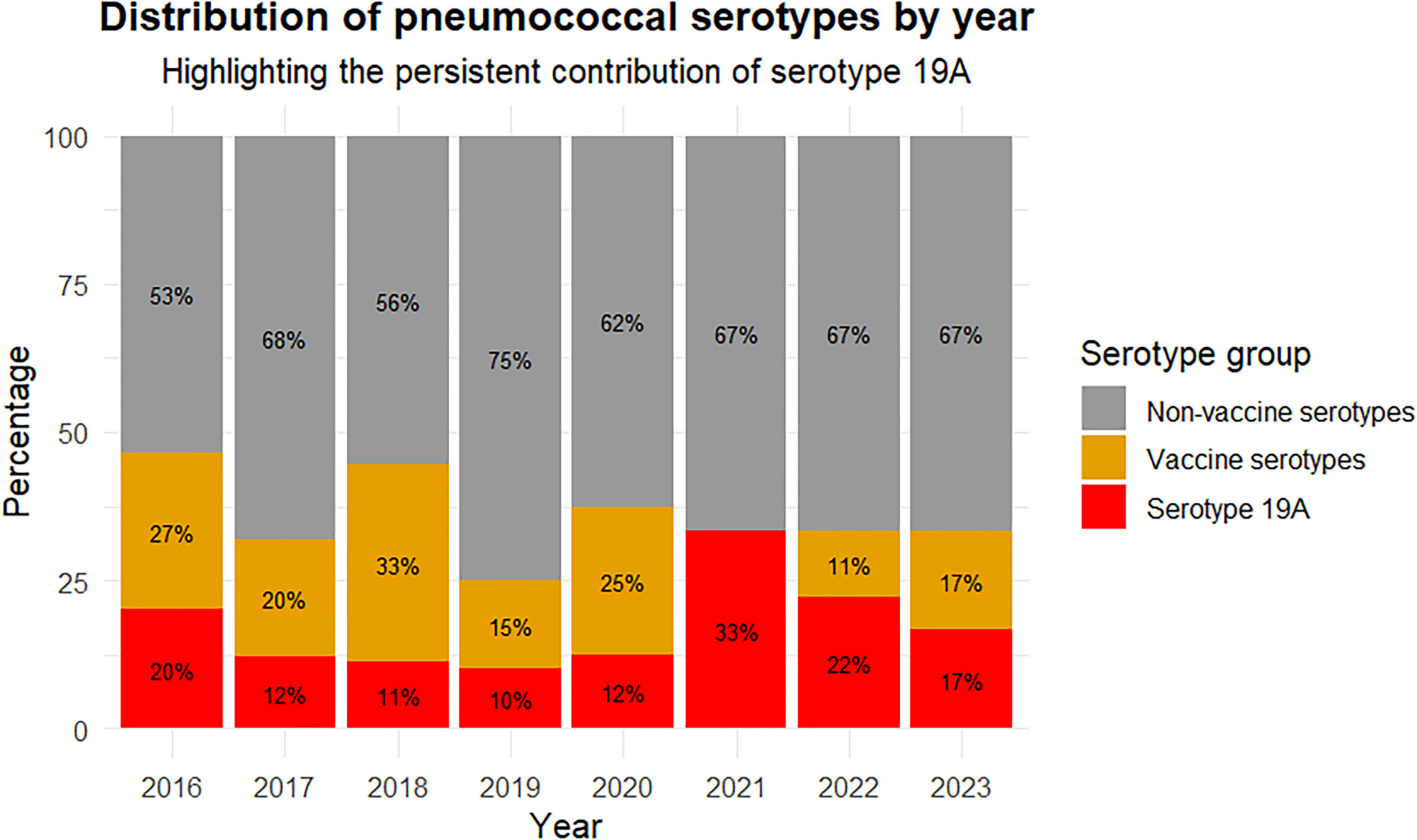
Annual proportional distribution of vaccine and non-vaccine pneumococcal serotypes causing IPD in adult and pediatric patients from two hospitals in Chile between 2016 and 2023. Stacked bar chart showing the yearly percentage of isolates classified as serotype vaccine, non-vaccine, and serotype 19A. The x-axis represents the years, and the y-axis represents the percentages of vaccine (in orange) and non-vaccine serotypes (gray), as well as serotype 19A (in red).

As shown in Figure 4, vaccine serotypes (orange) covered by PCV13 (introduced in PNI in November 2017) and PPSV23 showed a clear declining trend throughout the study. In contrast, non-vaccine serotypes (Figure 4, in gray) remained at high levels despite fluctuations. In fact, non-vaccine serotypes have been the most frequently isolated among all serotypes (Figure 4).

Vaccine serotype 19A (Figure 4, in red) consistently showed a low but steady presence throughout the period studied.

### 3.5 Comparison of antibiotic susceptibility of *S. pneumoniae* serotypes 19A and non-19A isolated from hospitalized patients with IPD between 2016 and 2023 in Chile

**FIGURE 5:**
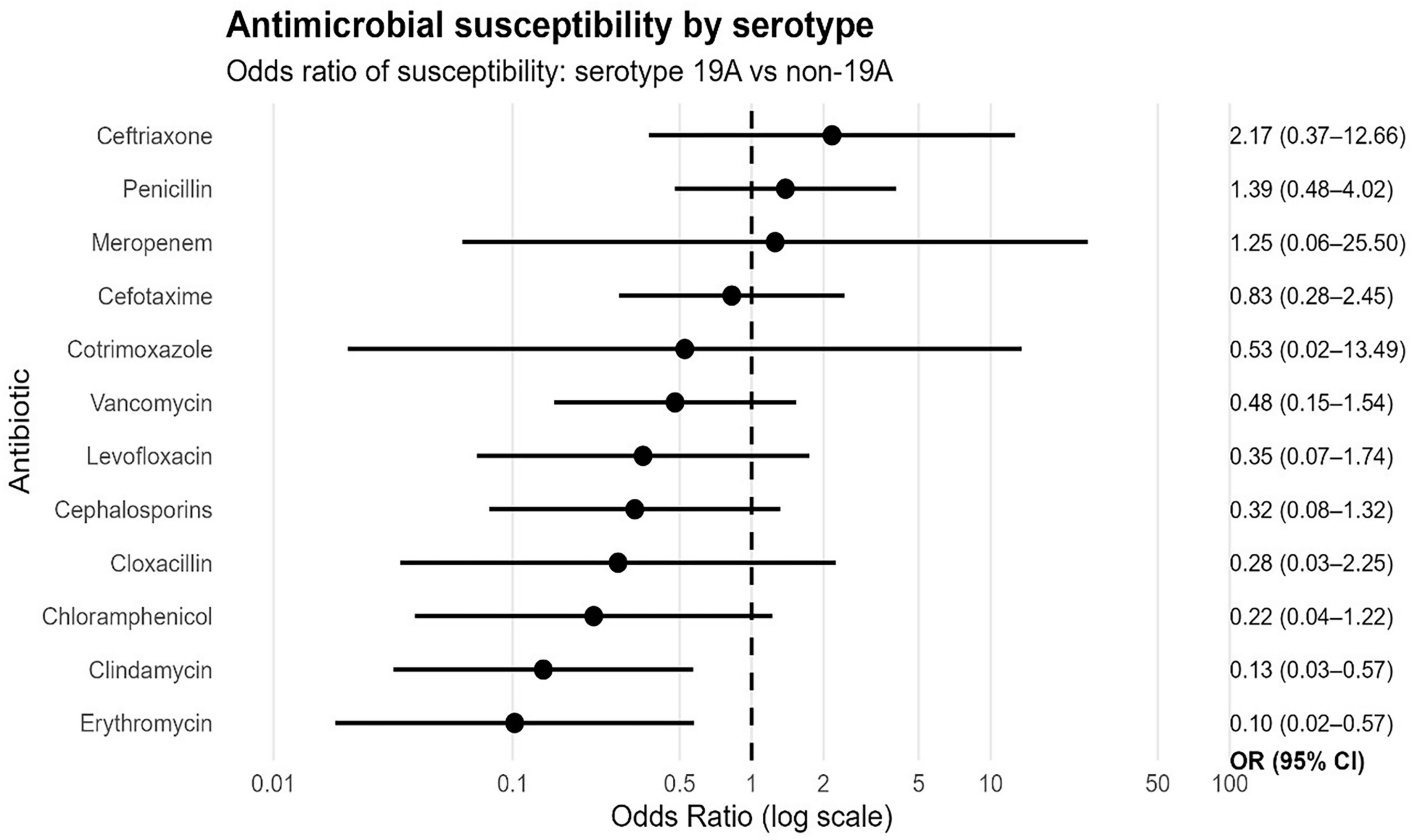
Isolates from adult and pediatric patients with IPD from two hospitals in Chile between 2016 and 2023. The dots represent odds ratios (OR) of antimicrobial susceptibility, and the horizontal lines represent their 95% confidence intervals, on a logarithmic scale. An OR greater than 1 indicates susceptibility to the tested antibiotic by the serotype or group of serotypes analyzed.

Antimicrobial susceptibility profiles were analyzed in *Streptococcus pneumoniae* serotypes isolated from hospitalized patients with IPD in Chile between 2016 and 2023 (Figure 5 and Table 2). Notably, a high proportion of susceptibility was observed for most of the antimicrobials tested (Table 2). Specifically, susceptibility to cefotaxime, vancomycin, levofloxacin, and chloramphenicol was 100%. Similarly, susceptibility to ceftriaxone reached 94.7%, with a resistance rate of 5.3%. Furthermore, susceptibility to penicillin was 52.8%, with an intermediate susceptibility rate of 2.8% and a resistance rate of 10.2%. On the other hand, low susceptibility to erythromycin (0.9%) and resistance to clindamycin (42.9%) were observed. Finally, susceptibility to meropenem was 50% with a similar proportion of resistance (50%).

**TABLE 2:** Antimicrobial susceptibility profile of *Streptococcus pneumoniae* in isolates from invasive pneumococcal disease patients of two hospitals of Chile between 2016 and 2023.

Characteristic	Susceptibility (%)		
Antibiotic	Susceptible	Intermediate	Resistant
Penicillin	52.8	2.8	10.2
Vancomycin	100	0	0
Cefotaxime	100	0	0
Cotrimoxazole	0.9	0	1.9
Erythromycin	0.9	0	0
Clindamycin	57.1	0	42.9
Levofloxacin	100	0	0
Cephalosporins	100	0	0
Meropenem	50	0	50
Ceftriaxone	94.7	0	5.3
Chloramphenicol	100	0	0
Cloxacillin	100	0	0

No intermediate sensitivity was observed for most of the antimicrobials tested, except for penicillin.

Antimicrobial susceptibility odds ratios (ORs) were estimated to compare *Streptococcus pneumoniae* isolates of serotype 19A with those of serotypes other than 19A (Figure 5 and Supplementary Table S4). Most antibiotics had ORs < 1, indicating a trend toward lower antimicrobial susceptibility in isolates of serotype 19A compared with those of other serotypes. This trend was particularly pronounced for erythromycin (OR 0.10; 95% CI: 0.02–0.57) and clindamycin (OR 0.13; 95% CI: 0.03–0.57), where statistically significant differences were observed when excluding the null value from their confidence intervals. Similarly, other antibiotics such as chloramphenicol (OR 0.22), cloxacillin (OR 0.28), cephalosporins (OR 0.32), and levofloxacin (OR 0.35) also showed ORs less than 1, indicating lower susceptibility by serotype 19A, although this difference did not reach statistical significance.

Conversely, some antibiotics showed ORs greater than 1, such as ceftriaxone (OR 2.17), penicillin (OR 1.39), and meropenem (OR 1.25), suggesting greater susceptibility by serotype 19A. However, in these cases, the confidence intervals included 1, indicating no statistically significant differences.

Overall, these results show a trend toward lower antimicrobial susceptibility by serotype 19A.

## 4 Discussion

Our retrospective, hospital-observational, cross-sectional surveillance analysis in two hospitals in Chile shows that non-vaccine serotypes of *S. pneumoniae* have been present between 2016 and 2023, causing IPD in both children and adults. We described clinical diagnoses, risk factors, and comorbidities among IPD patients during this period, along with gender, age, vaccination status, and serotype variation.

Data showed that in children, IPD affects preschoolers and school-age kids, while the disease is spread across all ages in adults (18 to over 65 years), with no notable difference between men and women in both children and adults. The likelihood of IPD was significantly higher in adults compared to children in this sample, which was directly related to hospital stays, as adults with IPD tended to stay longer (Table 1). This observation is consistent with previous studies showing that IPD tends to present with greater clinical severity and higher rates of complications in older people (Abdullahi et al., 2024; Henriques-Normark and Tuomanen, 2013; Weiser et al., 2018; Winje et al., 2021). Also, while respiratory infections were most common in children, adults showed a similar rate of respiratory and CNS infections, with no febrile syndromes observed in the adult group. Similar patterns have been described in other epidemiological studies of pneumococcal disease, where bacteremia pneumonia represents the predominant form of IPD in adults, while children may present a broader spectrum of clinical manifestations (Abdullahi et al., 2024; Alsina et al., 2015; Ozisik, 2025).

Globally, and particularly in Latin America, *S. pneumoniae* is known to cause asymptomatic nasopharyngeal carriage in approximately 20–40% of healthy adults, with even higher rates reported in pediatric populations (Lijek and Weiser, 2012; Prunas et al., 2024). Therefore, at-risk populations are often and persistently colonized by *S. pneumoniae*. The progression from carriage to disease depends on several risk factors, including age, inflammatory conditions, geographic location, socioeconomic status, genetics, and immune system health (Bogaert et al., 2004; Simell et al., 2012).

Regarding risk factors, our results showed that adults exhibit a more diverse set of risk factors than children. The most common risk factor across all age groups was immunosuppression, affecting 15 children and 8 adults. These findings are consistent with previous reports indicating that comorbidities significantly increase the risk of developing severe IPD in adult populations (Croucher et al., 2011; Pichichero and Casey, 2007; Sohail et al., 2018).

The prevalence of *S. pneumoniae* causing IPD showed a similar pattern in both age groups: an initial increase until 2017, followed by a sharp decline in 2018 (Figure 1). This trend could be due to factors such as changes in vaccination policies (in 2017, PCV13 was introduced into the PNI of Chile to replace PCV10), immunization coverage, or shifts in pneumococcal serotype distribution. Additionally, in Chile, a significant event occurred in 2020: the start of the pandemic lockdown. In this country, this period began in March 2020 and ended in September 2022. The concurrent lowest values observed in both groups (adults and children) in 2021 may also reflect the cumulative effect of more effective control measures.

Currently available *S. pneumoniae* vaccines protect against IPD by inducing anti-capsular opsonic antibodies to eliminate *S. pneumoniae* serotypes in the vaccine (Brueggemann et al., 2007; Song et al., 2013). However, the removal of vaccine serotypes has led to the emergence of non-vaccine serotypes that colonize and cause disease in their place (Croucher et al., 2011; Pichichero and Casey, 2007).

It is well known that vaccination significantly reduces the burden of infectious diseases (Andre et al., 2008). However, effective vaccines not only protect those vaccinated but also decrease disease prevalence among unvaccinated individuals in the community through “indirect effects” or “herd protection” (Ali et al., 2005). This study showed low vaccination rates among adults (6.3%), compared to a much higher rate among infants (71.4%). In this case, the wide confidence interval suggests some degree of imprecision, likely due to the relatively small sample size. However, the magnitude and consistency of the association support a marked difference in vaccination coverage between the age groups (Table 1). This event correlates with the high prevalence and frequency of serotypes found in the IPD patients studied, as well as the high death rate among adults (20.4%). Notably, vaccine-serotype 19A was the only prevalent serotype during the study period. The greater potential to cause IPD than other serotypes could be due to its high carriage rates and capsular switching, which involves genetic recombination that leads to a new capsular serotype (Brueggemann et al., 2007). Adults exhibit less serotype diversity, with high frequencies. Meanwhile, some serotypes common in children, such as non-vaccine serotype 24F, are absent in adults, indicating that age influences serotype distribution and IPD occurrence. Statistical analysis revealed significant differences in *S. pneumoniae* serotype distribution between children and adults with IPD (χ^2^ = 65.19, *p* < 0.001), suggesting that the prevalence of each serotype varies substantially between both groups, indicating a differentiated epidemiology of IPD-causing serotypes by age. As shown in Figure 3, children more frequently harbor serotypes 19A, 24F, 6C, 3, 23B, and 35B, while adults more often carry 3, 23A, 23B, 19A, and 6C. The PPV23 and PCV13 vaccines protect against only two of the more common serotypes found in both groups: 3 and 19A, highlighting the urgent need to update vaccine serotype coverage. Moreover, this high prevalence of serotype 3 in the studied adult cohort coincides with the global landscape and may be due to its unique capsular properties that evade vaccine-induced immunity (El Zein et al., 2025). Interestingly, while the introduction of PCV13 has generally affected the distribution of *S. pneumoniae* serotypes causing IPD, the data from this study show that serotype 19A remains the most prevalent among children (21.8%). This is consistent with recent evidence indicating that the impact of 19A has varied across regions following PCV13 implementation and often persists despite high immunization coverage (El Zein et al., 2025).

The results presented in this study are broadly consistent with the latest ISP bulletin on the surveillance of *S. pneumoniae* causing IPD in the Chilean population from 2012 to 2023 (Ministerio de Salud de Chile. Instituto de Salud Pública, 2024), which showed that the most common serotypes during that period were serotypes 3 (13.5%), followed by 19A (12.0%), with an increasing contribution of these two serotypes over the evaluated period (Rodgers et al., 2021).

Overall, a significant proportion of isolates remained sensitive to first-line antibiotics (Figure 5, Table 2, and Supplementary Table S4), consistent with findings from IPD cases in other parts of the world (Zhanel et al., 2023). Recent evidence has shown that the implementation of the PCV13 vaccine has been associated with changes in the antimicrobial susceptibility landscape, as well as shifts in the distribution of circulating serotypes of *S. pneumoniae* causing IPD (Huang et al., 2023; Llorente et al., 2026; Vermeulen et al., 2025). Nevertheless, this effect remains heterogeneous, with stable or increasing resistance patterns to penicillin and erythromycin reported in various settings (Centers for Disease Control and Prevention, 2019). In this context, the trend observed in this study toward lower susceptibility in serotype 19A compared to non-19A serotypes could reflect the persistence of a lineage historically associated with resistance in the post-PCV13 period. These processes likely contribute to the specific antimicrobial susceptibility profiles observed locally. Moreover, the emergence of non-vaccine serotypes such as 24F is particularly concerning. This phenomenon, known as “vaccine-driven selection,” favors the expansion of clones that not only escape vaccine pressure but also carry resistance genes, potentially complicating empirical treatment guidelines for IPD in Chile.

Taken together, the findings presented here support the concept that selective vaccination pressure reduces the prevalence of covered serotypes but enables the growth of those without immunity, thus filling the ecological niche (Flasche et al., 2013; Masala et al., 2017).

Our findings provide an exploratory overview of the evolution of *S. pneumoniae* serotypes isolated from patients with IPD at two Chilean hospitals, including the period following the implementation of the PCV13 vaccine in that country. The persistence of serotypes 3 and 19A, along with the emergence of other serotypes not included in the vaccine, such as 24F and 23B, suggests a dynamic process of serotype replacement that requires close attention. While these results are specific to these two hospitals and the study period, they highlight the importance of maintaining high-resolution laboratory surveillance and the need for continuous assessment of the epidemiological situation of *S. pneumoniae* serotypes causing IPD in each country. This would allow health authorities to decide whether to introduce different pneumococcal vaccines into their national immunization programs. In particular, these data should be considered as preliminary evidence to encourage national studies that will provide a more definitive basis for future updates to pneumococcal vaccination strategies in Chile.

Regarding representativeness, it is important to note that our data comes from two major hospitals in different geographical areas of Chile. While these findings cannot be directly generalized to the entire country, they offer a critical and detailed view of a region with unique demographic characteristics, including a significant migrant population. To ensure the reliability of this regional data, we conducted sensitivity analyses that confirmed the stability of our main results, thus reinforcing the internal validity of our findings within this specific geographical context.

Some limitations of this study include the representativeness of the sample and its size: patients are from specific hospitals (“Dr. Luis Calvo Mackenna” in Santiago is a pediatric hospital), which may not reflect the entire national population; the small number of cases may limit the ability to conduct detailed statistical analysis (e.g., subgroups by age, risk factors). Another limitation observed is incomplete data on clinical variables, such as Nationality, ICU admission, or vaccination status, which may limit the interpretation of risk factors. This limitation was addressed by conducting sensitivity analyses to account for potential bias arising from missing clinical records. The consistency of the findings across these scenarios reinforces the reliability of our observation regarding the high burden of disease in the studied cohort, despite the inherent limitations of a retrospective design.

Another potential limitation of this study is the absence of a formal baseline comparison prior to vaccination. Nonetheless, the 2016–2023 timeframe was intentionally selected to evaluate the dynamics of *S. pneumoniae* during the period of highest PCV13 selective pressure. This allowed for the robust identification of non-vaccine serotypes that have emerged as primary drivers of IPD burden in the current epidemiological context, providing a high-resolution overview of the contemporary challenges for public health policies in the region. Despite these limitations, this study provides valuable information on the current epidemiology of IPD in Chile, highlighting the importance of sustained microbiological surveillance of this pathogen and the need to constantly evaluate evolving vaccination strategies.

## Data Availability

The original contributions presented in the study are included in the article/Supplementary material, further inquiries can be directed to the corresponding author.
