# Supplementary material for: Change in the distribution of Streptococcus pneumoniae serotypes causing invasive pneumococcal disease among pediatric and adult patients in Chile between 2016 and 2023
Source: Front Microbiol. 2026 May 14;17:1819434. doi: 10.3389/fmicb.2026.1819434 (PMC13218216; doi:10.3389/fmicb.2026.1819434)
Supplement: Supplementary file 1 [file Data_Sheet_1.pdf]

Supplementary Material

1 Supplementary Figures and Tables

S1 Table. *S. pneumoniae* serotype distribution among IPD pediatric patients from the Dr. Luis Calvo Mackenna Hospital between 2016-2023.

Patient code	Admission (Year)	Gender	Range of age (Years)	Serotype
33-P	2016	F	1–4	20
35-P	2016	F	1–4	19A
53-P	2016	F	5–17	7F
6-P	2017	F	>1	22F
11-P	2017	F	>1	6A
18-P	2017	M	1–4	19A
21-P	2017	M	1–4	3
28-P	2017	M	1–4	24F
34-P	2017	F	1–4	19A
40-P	2017	M	5–17	16F
43-P	2017	M	5–17	19A
45-P	2017	M	5–17	12F
50-P	2017	F	5–17	15B
39-P	2018	M	1–4	19A
51-P	2018	M	5–17	6C
10-P	2019	M	>1	15A
12-P	2019	M	1–4	19A
16-P	2019	F	1–4	24F
17-P	2019	F	1–4	6C
26-P	2019	M	1–4	15A
42-P	2019	F	5–17	24F
44-P	2019	F	5–17	24F
48-P	2019	F	5–17	7F

7-P	2021	F	>1	6C
8-P	2022	M	>1	24F
15-P	2022	M	1–4	18A
20-P	2022	M	1–4	11B
5-P	2023	M	>1	3
22-P	2023	F	1–4	9N
49-P	2023	M	5–17	23A
57-P	2023	M	5–17	23A

Abbreviations: F: Female; M: Male; Grouped ages as: >1, 1–4, 5–17 years.

S2 Table. ***S. pneumoniae*** **serotype distribution among IPD pediatric patients from the Regional Antofagasta Hospital between 2016-2023.**

Patient code	Admission (Year)	Gender	Range of age (Years)	Serotype
4-P	2016	F	>1	19A
9-P	2016	F	>1	19A
13-P	2016	M	1–4	3
23-P	2016	M	1–4	19A
36-P	2016	F	1–4	34
37-P	2016	M	1–4	35B
24-P	2017	F	1–4	24F
38-P	2017	M	1–4	9N
52-P	2017	M	5–17	6C
29-P	2019	F	1–4	3
30-P	2019	M	1–4	23B
14-P	2020	F	1–4	35B
55-P	2020	M	14	19A
46-P	2022	F	5–17	35B
47-P	2022	M	5–17	19A
31-P	2023	M	1–4	24F
41-P	2023	M	5–17	19A
2-P	2024	F	>1	12F

Abbreviations: F: Female; M: Male; Grouped ages as: >1, 1–4, 5–17 years.

S3 Table. ***Streptococcus pneumoniae*** **serotype distribution among IPD adult patients from the Regional Antofagasta Hospital between 2016-2023.**

Patient code	Admission (Year)	Gender	Range of age (Years)	Serotype
59-A	2016	M	18–64	34
66-A	2016	F	18–64	48
71-A	2016	M	18–64	17F
77-A	2016	F	18–64	6A
86-A	2016	M	18–64	4
94-A	2016	M	≥65 years	12F
58-A	2017	F	18–64	12F
60-A	2017	M	18–64	4
62-P	2017	F	18–64	3
67-A	2017	M	18–64	23B
74-A	2017	M	18–64	19F
79-A	2017	F	18–64	23B
80-A	2017	M	18–64	22F
87-A	2017	M	18–64	48
92-A	2017	M	18–64	10A
98-A	2017	M	≥65 years	18A
102-A	2017	M	≥65 years	35F
104-A	2017	F	≥65 years	15A
56-A	2018	M	18–64	4
61-A	2018	M	18–64	3
65-A	2018	M	18–64	7C
78-A	2018	F	18–64	10A
95-A	2018	F	≥65 years	23A
96-A	2018	M	≥65 years	3
103-A	2018	F	≥65 years	6C
63-A	2019	M	18–64	35B
68-A	2019	M	18–64	19A

72-A	2019	F	18–64	23A
81-A	2019	M	18–64	20
84-A	2019	M	18–64	1
88-A	2019	M	18–64	11C
89-A	2019	M	18–64	23A
93-A	2019	M	18–64	23A
99-A	2019	F	≥65 years	6C
69-A	2020	F	18–64	9N
75-A	2020	F	18–64	10F
90-A	2020	M	18–64	3
91-A	2020	F	18–64	35F
101-A	2020	M	≥65 years	3
105-A	2020	M	≥65 years	6C
64-A	2021	M	18–64	19A
83-A	2021	M	18–64	10F
70-A	2022	M	18–64	23A
73-A	2022	F	18-64	15B
76-A	2022	M	18–64	3
97-A	2022	F	≥65 years	19A
85-A	2023	F	18–64	23B
100-A	2023	M	≥65 years	6B

Abbreviations: F: Female; M: Male; Grouped ages as: 18–64, and ≥65 years.

S4 Table. Comparison of antimicrobial susceptibility of S. pneumoniae serotype 19A vs non-19A causing IPD, 2016–2023.

Antibiotic	19A (%)	Non-19A (%)	*p*-value*	*p*-adjusted*
**Penicillin**	46.7	55.1	0.59	0.78
**Vancomycin**	73.3	55.1	0.26	0.53
**Cefotaxime**	40	36	0.78	0.93
**Cotrimoxazole**	0	1.1	1	1
**Erythromycin**	20	2.2	0.021	0.12
**Clindamycin**	26.7	4.5	0.014	0.12
**Levofloxacin**	13.3	5.6	0.26	0.53
**Cephalosporins**	20	7.9	0.15	0.47
**Meropenem**	0	3.4	1	1
**Ceftriaxone**	6.7	18	0.45	0.68
**Chloramphenicol**	13.3	3.4	0.15	0.47
**Cloxacillin**	6.7	2.2	0.38	0.64

*The Fisher exact test.
