# Supplementary material for: Change in the distribution of Streptococcus pneumoniae serotypes causing invasive pneumococcal disease among pediatric and adult patients in Chile between 2016 and 2023
Source: Front Microbiol. 2026 May 14;17:1819434. doi: 10.3389/fmicb.2026.1819434 (PMC13218216; doi:10.3389/fmicb.2026.1819434)
Supplement: Supplementary file 2 [file Data_Sheet_2.pdf]

STROBE Statement—Checklist of items that should be included in reports of ***cross-sectional studies***

	Item No	Recommendation	Relevant text from manuscript
**Title and abstract**	1	(*a*) Indicate the study's design with a commonly used term in the title or the abstract	Abstract describes: "This is a retrospective, hospital-observational, cross-sectional study."
		(*b*) Provide in the abstract an informative and balanced summary of what was done and what was found	**Methods** and **Results** in the abstract provides an informative and balanced summary of what was done and what was found.
**Introduction**			
Background/rationale	2	Explain the scientific background and rationale for the investigation being reported	**Introduction** includes an explanation of the scientific background and rationale for the investigation.
Objectives	3	State specific objectives, including any prespecified hypotheses	The **introduction** states: "This study aims to…", explaining specific objectives and hypotheses.
**Methods**			
Study design	4	Present key elements of study design early in the paper	**Materials and Methods,** key aspects of the study design are described. We have made every effort to provide all information necessary for reproducibility and to ensure the analysis is precise.
Setting	5	Describe the setting, locations, and relevant dates, including periods of recruitment, exposure, follow-up, and data collection	**Materials and Methods** section, **Settings and Participants**, contains a description of the locations.<br>**Materials and Methods** section, **Data collection and eligibility criteria** describes the relevant dates and data collection.
Participants	6	(*a*) Give the eligibility criteria, and the sources and methods of selection of participants	**Materials and Methods,** section **Data collection and eligibility criteria** describes the eligibility criteria, and the sources and methods of selection of participants
Variables	7	Clearly define all outcomes, exposures, predictors, potential confounders, and effect modifiers. Give diagnostic criteria, if applicable	**Materials and Methods** section, **Data collection and eligibility criteria** define outcomes, predictors, effect modifiers, and other variables.
Data sources/ measurement	8*	For each variable of interest, give sources of data and details of methods of assessment (measurement). Describe comparability of assessment methods if there is more than one group	In the **Materials and Methods** section, **Data collection and eligibility criteria,** sources of data, and details of measurements are given. All data were sourced from each patient's clinical data, combining information on IPD diagnosis and serotype confirmation.
Bias	9	Describe any efforts to address potential sources of bias	In the **Materials and Methods** section, **Data collection and eligibility criteria**, efforts to address potential sources of bias are described: "To reduce selection bias…"
Study size	10	Explain how the study size was arrived at	In the **Materials and Methods** section, **Data collection and eligibility criteria** includes the eligibility criteria for this observational retrospective study.<br>The sample size corresponds to the total number of confirmed cases in both hospitals during the period, so no prior sample size calculation was performed.
Quantitative variables	11	Explain how quantitative variables were handled in the analyses. If applicable, describe	In the **Materials and Methods** section**, Data collection and eligibility criteria** describes all quantitative variables taken into account for

		which groupings were chosen and why	analysis.<br>In the **Materials and Methods** section, **the Statistical analysis** describes the handling of quantitative data.<br>We have made every effort to provide comprehensive management of all quantitative variables.
Statistical methods	12	(*a*) Describe all statistical methods, including those used to control for confounding	In the **Materials and Methods** section, **Statistical analysis** describes all statistical methods.
		(*b*) Describe any methods used to examine subgroups and interactions	**Not applicable:** This is an observational study of all reported cases during 2016 and 2023.
		(*c*) Explain how missing data were addressed	In the **Materials and Methods** section, **Data collection and eligibility criteria** states that all data from each patient's clinical records were obtained by cross-referencing information from the IPD diagnosis and serotype confirmation. Patients without information on the diagnosis of IPD or serotype confirmation were not included in this study.<br>We have made every effort to extract all information, even from physical medical records, from each hospital.<br>All missing data are included as "Without Information" (W.I.) in **Table 1.**
		(*d*) If applicable, describe analytical methods taking account of sampling strategy	**Not applicable:** This is an observational study of all reported cases during 2016 and 2023.
		(*e*) Describe any sensitivity analyses	A sensitivity analysis was performed comparing characteristics of the included and excluded cases, and the models were repeated, excluding patients with unrecorded vaccination status and incomplete comorbidities, to assess the robustness of the results.
**Results**			
Participants	13*	(a) Report numbers of individuals at each stage of study—eg numbers potentially eligible, examined for eligibility, confirmed eligible, included in the study, completing follow-up, and analysed	In **Table 1** report the confirmed number of data obtained from patients in this observational study. Supplementary Material, Tables 1, 2, and 3, report the number of individuals from each hospital. The sample size is the total number of confirmed cases across both hospitals during the period, so no prior sample size calculation was performed. *There are no exposed or unexposed groups since this is an observational retrospective study
		(b) Give reasons for non-participation at each stage	Cases with missing data on essential variables were excluded from the corresponding analyses.
		(c) Consider use of a flow diagram	
Descriptive data	14*	(a) Give characteristics of study participants (eg demographic, clinical, social) and information on exposures and potential confounders	In **Table 1** summarizes all characteristics of the data obtained from patients in this observational study.<br>*There are no exposed or unexposed groups since this is an observational retrospective study
		(b) Indicate number of participants with missing data for each variable of interest	In **Table 1,** missing data of patients is indicated as without information (W.I.) for every variable of interest.
Outcome data	15*	Report numbers of outcome events or summary measures	In **Results**, outcome events are reported. *There are no exposed or unexposed groups since this is an observational retrospective study
Main results	16	(*a*) Give unadjusted estimates	In **Results,** all statistical analyses and

		and, if applicable, confounder-adjusted estimates and their precision (eg, 95% confidence interval). Make clear which confounders were adjusted for and why they were included	unadjusted estimates are given.<br>*There are no exposed or unexposed groups since this is an observational retrospective study
		(*b*) Report category boundaries when continuous variables were categorized	In **Results,** the range of the data and mean or median values within each category of this observational study are reported.
		(*c*) If relevant, consider translating estimates of relative risk into absolute risk for a meaningful time period	**Not applicable:** This is an observational study of all reported cases during 2016 and 2023.
Other analyses	17	Report other analyses done—eg analyses of subgroups and interactions, and sensitivity analyses	**Not applicable:** This is an observational study of all reported cases during 2016 and 2023.
**Discussion**			
Key results	18	Summarise key results with reference to study objectives	In **Discussions,** includes key results with reference to the specific objective of this observational study.
Limitations	19	Discuss limitations of the study, taking into account sources of potential bias or imprecision. Discuss both direction and magnitude of any potential bias	In **Discussions**, final paragraph states: "Some limitations of this study include…" detailing all the limitations and potential bias found in this observational study.
Interpretation	20	Give a cautious overall interpretation of results considering objectives, limitations, multiplicity of analyses, results from similar studies, and other relevant evidence	In **Discussions**, interpretations of results are provided, considering the specific objective and limitations, and are compared with results and analyses from other similar studies.
Generalisability	21	Discuss the generalisability (external validity) of the study results	In **Discussions**, external validity of the study results are discussed.
**Other information**			
Funding	22	Give the source of funding and the role of the funders for the present study and, if applicable, for the original study on which the present article is based	In the **Acknowledgements** lists the sources of funding.

*Give information separately for exposed and unexposed groups.

Note: An Explanation and Elaboration article discusses each checklist item and gives methodological background and published examples of transparent reporting. The STROBE checklist is best used in conjunction with this article (freely available on the Web sites of PLoS Medicine at http://www.plosmedicine.org/, Annals of Internal Medicine at http://www.annals.org/, and Epidemiology at http://www.epidem.com/). Information on the STROBE Initiative is available at www.strobe-statement.org.
